# Prefabricated building construction in materialization phase as catalysts for hotel low-carbon transitions via hybrid computational visualization algorithms

**DOI:** 10.1038/s41598-025-92200-8

**Published:** 2025-03-05

**Authors:** Gangwei Cai, Xiaoting Guo, Yuguang Sun

**Affiliations:** 1 College of Architecture and Urban Planning, Tongji University, Shanghai, 200092 China; 2 Hangzhou International Urbanology Research Center & Zhejiang Urban Governance Studies Center, Hangzhou, 311121 China; 3 Baoye Daiwa Industrialized House Manufacturing Co., Ltd., Daiwa House Group, Osaka, 530-8241 Japan; 4 Baoye Group Company Limited, Shaoxing, 312030 China; 5 School of Civil Engineering and Architecture, Zhejiang University of Science and Technology, Hangzhou, 310023 China

**Keywords:** Carbon decoupling, Prefabricated building, Materialization phase, Sustainable tourism, Star-rated hotel, Machine learning spatio-temporal analysis, Sustainable development goals (SDGs), Environmental economics, Sustainability, Energy and society, Energy economics, Energy efficiency, Energy management, Energy policy, Political economy of energy

## Abstract

This study examines the carbon emissions of star-rated hotels in Hangzhou, comparing the environmental impact of prefabricated construction (PC) and conventional construction (CC) methodologies. The research reveals that PC generally results in lower carbon emissions during the materialization phase, with notable variations across different hotel star levels and administrative regions. Higher-star hotels exhibit higher total emissions, primarily due to larger scale and reliance on conventional construction methods. In contrast, lower-tier hotels benefit more consistently from the adoption of prefabricated construction, leading to significant reductions in carbon emissions. Regional analysis shows that the impact of the COVID-19 pandemic on hotel turnover and carbon decoupling trends varies, with core urban areas experiencing a more pronounced decoupling effect, while suburban regions exhibited slower recovery. The findings underscore the potential for prefabricated construction to reduce carbon footprints, particularly in mid-tier and lower-tier hotels. This study contributes to the understanding of sustainable construction practices in the hotel industry and provides a foundation for future research focused on refining carbon emission assessments, incorporating real-world data, and exploring the integration of renewable energy and lifecycle emissions.

## Introduction

The construction industry is a significant contributor to global carbon emissions, with buildings accounting for a substantial portion of resource consumption and environmental degradation^1^. Global hospitality faces significant sustainability challenges, particularly in relation to SDGs 7.3, 8.9, 9.4, 11.3, and 12.5^2^. In the hospitality sector, where the demand for new constructions is steadily increasing, understanding the environmental impacts of various construction methods is critical for developing sustainable practices^3^. Among the available construction technologies, prefabricated construction (PC) has gained attention due to its potential to reduce carbon emissions and improve construction efficiency^4^. However, while substantial research has focused on the carbon footprint of conventional construction methods, there remains a gap in understanding how modern prefabricated techniques compare across different building sectors, particularly within the context of hotel construction^5^. This study aims to address this gap by assessing the carbon emissions of star-rated hotels in Hangzhou, China, with a focus on comparing prefabricated construction (PC) and conventional construction (CC) methodologies. Specifically, the study investigates the carbon emissions associated with the materialization phase—encompassing the production, transportation, and construction stages of building materials—and explores how these emissions vary with hotel star ratings and regional differences. Given the rapid urbanization of Hangzhou and its role as a major tourist hub, it is essential to assess the environmental impact of hotel construction in the region, considering both the global shift toward more sustainable practices and the local context. This study also examines the decoupling of economic growth and environmental impact, particularly in light of recent global events such as the COVID-19 pandemic, which had a significant effect on hotel turnover and carbon emissions^6^. Using the Tapio decoupling model^7,8^, the research investigates how various regions in Hangzhou experienced different decoupling effects, with particular attention to the role of construction methods in these trends. Through this research, we aim to provide a comprehensive comparison of prefabricated and conventional construction methods, focusing on their environmental implications for hotel buildings^9^. The findings are expected to offer insights into the potential for reducing carbon emissions in the hotel sector, particularly in mid-tier and lower-tier hotels, and contribute to the broader discourse on sustainable building practices in urban environments.

The tourism sector is a significant contributor to global carbon dioxide (CO2) emissions, with various sub-sectors such as aviation, road transport, and accommodation each playing a notable role^10^. As international tourist arrivals continue to grow, the associated greenhouse gas (GHG) emissions are expected to rise as well, with hotel accommodation becoming a key non-transport source of emissions within the tourism industry^11^. The hospitality sector’s carbon emissions are projected to increase significantly, highlighting the urgent need for sustainable tourism practices aimed at minimizing CO2 emissions^12^. In recent years, there has been an increasing focus within the academic community on low-carbon and sustainable tourism, with research often examining the measurement, evolution, and drivers of carbon emissions, as well as their relationship with the broader tourism economy^13,14^. While there are variations in research methodologies and focus areas, the hotel industry is typically studied as a major segment of the tourism sector^15^. Scholars frequently calculate the annual carbon emissions of the hotel industry by considering indicators such as occupancy rates, energy consumption, number of hotel beds, and the duration of a year^16^. These metrics are commonly used for operational carbon emission calculations in the hotel industry^17^. Although operational energy consumption accounts for the largest portion of the hotel industry’s overall carbon footprint throughout its lifecycle, the construction phase still contributes a significant share^18^. Policies targeting energy efficiency and environmental protection have effectively reduced emissions during the operational phase^19^. However, the intensity and concentration of emissions during the construction phase warrant further investigation into strategies for reducing carbon emissions in this critical period^20^. Despite the increasing focus on operational emissions, the materialization phase remains underexplored^21^. This is due to challenges in obtaining relevant data, limited tools for assessing embodied carbon, and the relative ease of collecting operational data compared to construction-related emissions^22^. Moreover, there are notable gaps in life-cycle assessment tools that can account for material waste, energy consumption, and carbon emissions during the construction phase^23^. This study aims to address these gaps and explore the carbon emissions associated with the materialization phase of hotel construction, with a focus on identifying potential strategies to reduce emissions during this crucial period.

The whole life cycle of a building encompasses all stages from raw material extraction to the demolition and disposal of the building, with greenhouse gas emissions occurring at each stage^24^. Scholars commonly divide the life cycle into four phases: building materials production and transportation, building construction, building operation, and building dismantling and recycling, with the first two stages collectively referred to as the materialization phase^25^. Since there is no universally accepted carbon emission calculation method specifically for prefabricated buildings, scholars have summarized three key carbon emission accounting methods: the material balance method, the actual measurement method, and the emission factor method, all of which draw on conventional building carbon emission accounting techniques^26^. While the emission factor method is widely adopted due to its simplicity, clarity, and the availability of established accounting formulas, activity data, and emission factors, it is important to acknowledge its limitations, particularly in the context of prefabricated buildings^27^. Specifically, the accuracy of emission factors for various materials used in prefabricated construction can significantly affect the reliability of the assessment^28^. For instance, variations in emission factors for materials such as prefabricated floor panels, walls, and insulation could lead to discrepancies in overall carbon emission estimates^29^. Additionally, the application of these methods to the hotel sector faces unique challenges, including the limited availability of hotel-specific activity data, the lack of comprehensive emission factor databases tailored to the hotel industry, and the variability of materials and construction methods used in hotel buildings^30^. Although research on prefabricated buildings has mainly focused on residential and office buildings, the distinct characteristics of hotel buildings, such as the inclusion of specialized facilities like commercial kitchens and large HVAC systems, necessitate further adaptation and refinement of carbon emission calculation methods^31^.

Prefabricated buildings are those in which components are prefabricated or semi-fabricated in a factory and transported to the construction site for installation and connection^32^. Research comparing conventional building technology with prefabricated construction has highlighted several advantages of the latter, including energy savings, emission reductions, waste minimization, shortened construction times, immunity to adverse weather conditions, and improved quality control^33^. As a result, prefabricated technology is often applied in energy-saving and emission-reducing initiatives, particularly in the construction of low-carbon cities^34^. While prefabricated technology has been widely used in residential and office sectors, its application in hotel buildings remains less studied^35^. However, existing research suggests that prefabricated construction offers high efficiency, controllable quality, and adaptability over conventional construction methods, with the potential to reduce a large carbon emission in the pre-construction phase^36^. Hotel buildings, while similar to residential buildings in terms of certain operational activities, differ in their complexity, particularly due to the inclusion of commercial kitchens, large HVAC systems, and extensive public spaces^37^. These unique features present challenges for applying prefabricated technology in the hotel sector, which may affect the feasibility and efficiency of prefabrication methods^38^. Although carbon emission calculations for hotel buildings can benefit from insights drawn from residential buildings, the economic and operational differences between the two types of buildings must be considered^39,40^. Hotels, as commercial premises, focus on profitability and brand-specific designs, often requiring customization that may not align with the standardization benefits of prefabrication^41,42^. Therefore, a deeper exploration of how these economic and design constraints influence the viability of prefabrication in the hotel sector is needed to better understand its potential for reducing carbon emissions^43^.

The term "decoupling," originally coined in the field of physics, has been widely adopted by scholars to describe the relationship between economic development and environmental pressure^44^. Currently, two main decoupling models are used to analyze the link between economic growth and carbon emissions: the Tapio model and the OECD model^8^. The Tapio decoupling model is particularly useful in analyzing the relationship between tourism carbon emissions and the tourism economy^45^. Compared to the OECD model, the Tapio model avoids the instability associated with selecting a base period and offers the advantage of identifying multiple types of decoupling states based on elasticity values^46^. According to the Tapio decoupling model, the decoupling relationship can be categorized into three states—negative decoupling, decoupling, and connection—based on elasticity coefficients, and further subdivided into eight grades, including weak negative decoupling, strong negative decoupling, expanding negative decoupling, weak decoupling, strong decoupling, declining decoupling, declining connection, and expanding connection, depending on the strength of these relationships^47^. While research on decoupling in the tourism sector has been extensive, the hotel industry, an important source of carbon emissions within the tourism economy, remains underexplored^48^. The application of decoupling models, particularly the Tapio model, to the hotel construction sector is significant, given the sector’s substantial contribution to environmental impacts^49^. Although there is growing research on the decoupling relationship in broader construction sectors, such as the general building industry, the hotel construction sector presents unique challenges and opportunities that require a tailored approach^50^. For instance, the hotel industry is often subject to more complex operational demands compared to general residential or office buildings, including the need for energy-intensive facilities such as commercial kitchens, large HVAC systems, and extensive public spaces, which can influence its carbon emission profile^51^. Thus, applying the Tapio model to assess the decoupling of hotel construction-related carbon emissions from economic growth is particularly relevant^52^. This model allows for a deeper understanding of how the hotel construction sector’s carbon emissions can be mitigated as the industry grows, making it crucial for sustainable tourism development^53^. Despite the lack of direct studies on decoupling in the hotel industry, the application of the Tapio model to this sector could offer valuable insights and guide future research on sustainable practices in hotel construction^54^.

## Methodology

Hybrid computational visualization algorithms were employed in this study can be classified into four major categories of computational and analytical approaches commonly used in environmental and sustainability research. Statistical and data visualization methods include scatter plot analysis, which falls under descriptive statistics and exploratory data analysis (EDA) and is used to visualize relationships between hotel star levels and carbon emission differences between conventional construction (CC) and prefabricated construction (PC)^55,56^. Additionally, decoupling analysis is conducted to examine economic-environmental decoupling trends through elasticity analysis and time-series statistical modeling, assessing the relationship between hotel turnover and carbon emissions. Machine learning and clustering algorithms are applied through K-Means clustering, an unsupervised machine learning technique widely used in pattern recognition, classification, and data segmentation, which enables the grouping of hotels into clusters based on their carbon emission profiles, revealing distinct emission characteristics across hotel categories. Spatiotemporal analysis and geographic data science methods include spatiotemporal decoupling analysis, leveraging geospatial data analytics and time-series modeling to understand the evolution of carbon emissions and economic activity across different regions. This approach, closely related to geographic spatial econometrics, evaluates the impact of external events such as COVID-19 and the Asian Games on emissions at various geographic scales. Lastly, environmental and carbon footprint assessment is performed using carbon emission calculations based on literature synthesis, adhering to the principles of life cycle assessment (LCA) and environmental impact assessment (EIA). Given the absence of a unified carbon emission standard, this study integrates findings from previous literature to establish an empirical foundation for emissions estimation. Python (version 3.8, https://www.python.org/) and EXCEL (LTSC20021, https://www.microsoft.com/zh-cn/microsoft-365/get-started-with-office-2024) were used in the current study.

**Fig. 1:**
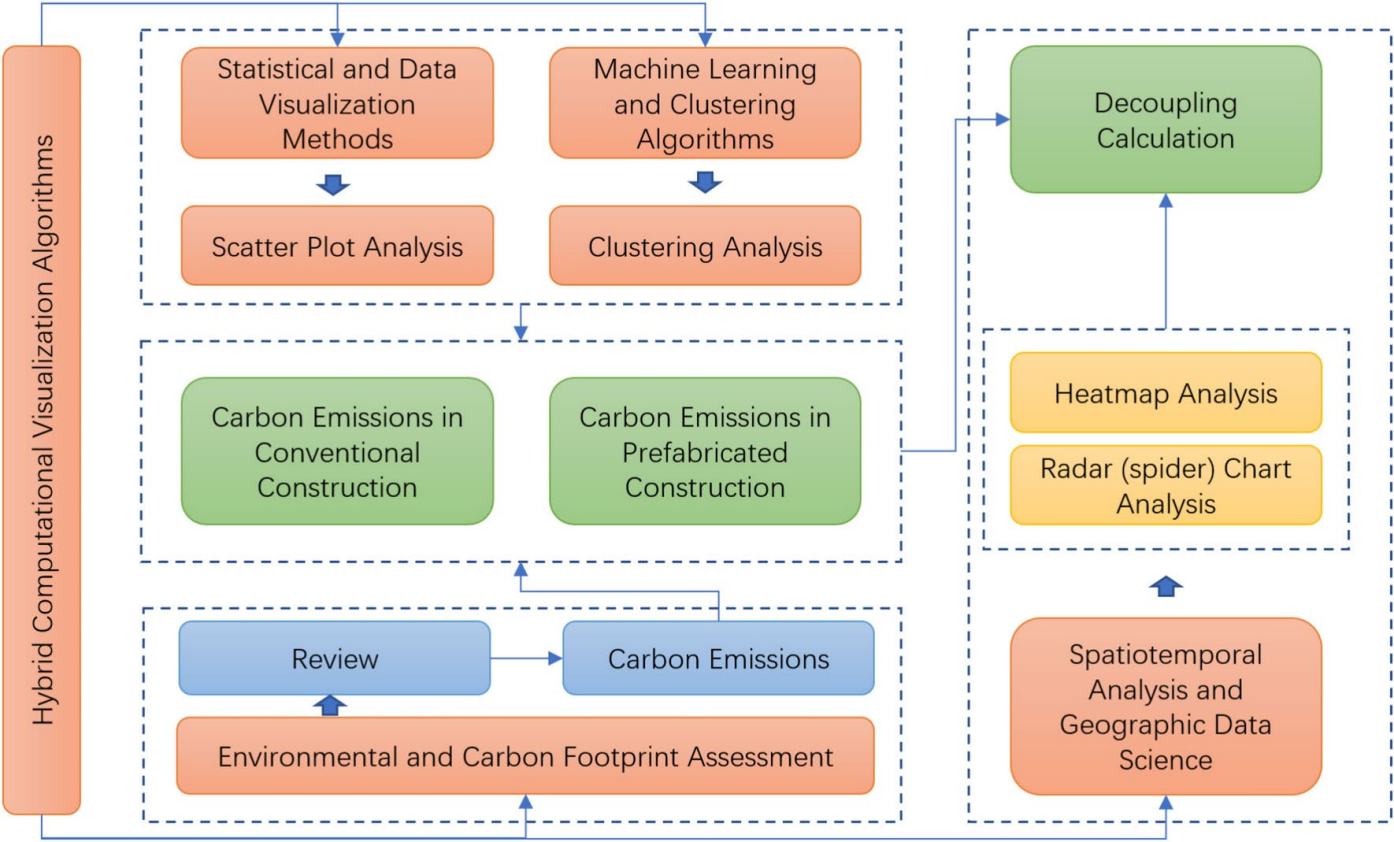
Research roadmap.

These combined methodologies provide a robust framework for analyzing the environmental impact of prefabricated construction in the hotel sector while accounting for regional and temporal variations in carbon. In this paper roadmap (Fig. 1), star-rated hotels within the Hangzhou city area are selected as the study cases, of which the core includes four districts, namely West Lake, Uptown, Gongshu and Binjiang, and the suburbs include six districts, namely Linping, Lin’an, Qiantang, Xiaoshan, Yuhang and Fuyang, as well as star-rated hotels in the three cities and counties of Jiande, Chun’an and Tonglu, excluding the one-star hotels. The study period is 2018–2023, spanning a total of five years before and after the epidemic and the Asian Games. The construction period, i.e., the materialization phase, as defined in this paper, includes the production of building materials, the transport of building materials, and the construction phase. To ensure a focused and comparable analysis, this study primarily considers the carbon emissions from the materialization phase of three commonly used materials—steel, concrete, and masonry—when evaluating the conventional construction mode. Other materials, such as insulation and glazing systems, are not included in the calculation, which is acknowledged as a limitation but allows for a clearer comparison of primary structural components. Regarding the prefabricated construction mode, a 100% prefabrication rate is assumed to isolate its impact on carbon emissions, specifically studying prefabricated floor slabs and prefabricated wall panels in the materialization phase. While this assumption may not fully reflect the diversity of real-world construction projects, it provides a theoretical upper boundary for the potential carbon reduction achievable through prefabrication. Future studies could refine this approach by considering hybrid construction methods and a broader range of materials to enhance applicability to practical scenarios.

### Methods

#### Carbon emission in the materialization phase of conventional construction method

The carbon emission in the materialization phase of conventional construction is mainly calculated and summarised by the production phase of building materials, the transport phase of building materials and the construction phase. The calculation formula is as follows:

1 $$\begin{array}{*{20}c} {{\text{C}} = C_{1} + C_{2} + C_{3} } \\ \end{array}$$

where C is the total carbon emissions in the conventional materialization phase; *C*_*1*_, *C*_*2*_ and* C*_*3*_ are respectively the carbon emission of materials production stage, materials transport stage, and materials construction stage.

2 $$\begin{array}{*{20}c} {C_{1} = \mathop \sum \limits_{i = 1}^{n} M_{i} EF_{i} } \\ \end{array}$$

where *C*_*1*_ is the carbon emission (kgCO_2_e) in the production stage of building materials; n is the number of types of building materials; EF_i_ denotes the carbon emissions factor (kgCO_2_e/unit of material consumption) of type i building materials; M_i_ is the consumption of building materials of category i. Since there are differences in the production methods of building materials and production energy consumption in different countries, this paper mainly focuses on summarising the research of Chinese scholars. According to the standard specification^57^ and literature collation, the carbon emission factor for concrete has been determined to be 364.00 kg/m^3^. For the block material, an aerated concrete block with a thickness of 250 mm has been selected, with its carbon emission factor established at 156.90 kg/m^3^. The carbon emission factor for steel reinforcement is set at 2208 kgCO_2_/t, as derived from the studies conducted by Huang, Liu and Li ^58,59^. Owing to the challenges in procuring specific volume data for hotels, this study employs a methodological approach that leverages the mathematical elegance of the Fibonacci sequence in the design of the hotel’s column model. Additionally, to accommodate the study’s requirements, the height of hotel floors is correlated with the incremental star rating, with the interval range of the floor height progressively increasing from 3 to 4.2 m.

3 $$\begin{array}{*{20}c} {C_{2}} = \mathop \sum \limits_{i = 1}^{n} M_{i} D_{i} T_{i} \\ \end{array}$$

where *C*_*2*_ is the carbon emission of building materials in the transport stage (kgCO_2_e); The variable n is the number of types of building materials; M_i_ is the consumption of building materials of type i; D_i_ is the transport distance of type i (km); and T_i_ is the carbon emission factor per unit of mass of transport distance under the transport mode of type i [kgCO_2_e/(t − km)]. The transport process mainly takes into account the transport process of transporting solid materials such as building materials, equipment and machinery to the construction site and transporting residual soil and solid construction waste out. The recycling coefficient of building materials is not considered in this paper. Conventionally, concrete and steel reinforcement constitute the principal materials that are transported, predominantly via road. Considering the common practice of trucks returning empty from construction sites, this paper adopts the return factor for empty trucks, *Fy*, as 1.67, based on the research conducted by Wang^60^.

The transport distance is assumed based on typical industry practices. Specifically, for one-way transport within the same city, a distance of 30 km is used, while for intercity transport, a distance of 150 km is assumed. The one-way transport distance for concrete is set at 30 km, whereas for reinforcement and masonry, it is set at 150 km^61^. Concrete is transported using concrete tankers with a capacity of 8 m^3^, while other materials are transported by 8-ton medium-sized diesel trucks. Regarding prefabricated components, the carbon emission factors are derived from previous studies. While these values provide a general reference, we acknowledge that prefabricated hotels may have unique characteristics that differ from the generic components considered in prior research. To address this limitation, we will explicitly discuss the potential differences and their implications in the revised manuscript. Additionally, we recognize that the assumed transport distances for prefabricated components may not fully capture the logistics specific to Hangzhou in 2023. The standard 150 km assumption was used due to the lack of publicly available real-world data on transportation distances for prefabricated hotel components. Future studies could improve accuracy by incorporating empirical data on transportation logistics for prefabricated buildings in different locations.

The density of diesel is 0.83–0.85 kg/L, 0.84 kg/L is taken, the carbon emission factor is taken as 0.179 (unit kgCO_2_e/(t − km)), and the amount of diesel consumed by the concrete tanker truck is taken as 0.54 (L/km)^59^.

4 $$\begin{array}{*{20}c} {C_{3} = \frac{{\mathop \sum \nolimits_{i = 1}^{n} E_{jz,i} EF_{i} }}{A}} \\ \end{array}$$

where *C*_*3*_ is the carbon emission of building materials in the construction stage (kgCO_2_/m^2^); The variable n is the number of types of building materials; E_jz,i_ is total energy consumption of type i of energy during the construction phase of the building (kWh or kg); and EF_i_ is the carbon emission factor of type i of energy (kgCO_2_/kWh or kgCO_2_/kg); A is the building area (m^2^). The carbon emissions during the construction phase are generated by the energy consumption of various mechanical and electrical equipment on the construction site, as well as the energy consumption of temporary buildings such as living quarters. However, due to the lack of data on actual energy consumption, shift numbers, and workload on the construction site^62^, this paper refers to the carbon emission data per square meter during the construction phase of residential buildings summarized by Song^63^. Based on the differences in hotel star ratings, the values are taken as 20.35–34.02 kgCO_2_. Therefore, the carbon emissions during the construction phase of the building are equal to the product of the total construction area and the carbon emissions per unit area during the construction phase.

#### Carbon emission in the materialization phase of prefabricated construction method

The carbon emissions of the prefabricated building materialization phase are primarily composed of emissions from three stages: the building material production stage, the building material transportation stage, and the construction stage. The calculation formula is as follow:

5 $$\begin{array}{*{20}c} {{\text{Y}} = Y_{1} + Y_{2} + Y_{3} } \\ \end{array}$$

where Y is the total carbon emissions in the materialization phase of prefabricated construction method; *Y*_*1*_*, Y*_*2*_ and* Y*_*3*_ are respectively the carbon emission of materials production stage, materials transport stage, and materials construction stage.

6 $$\begin{array}{*{20}c} {Y_{1} = \mathop \sum \limits_{i = 1}^{n} M_{i} EF_{i} } \\ \end{array}$$

where *Y*_*1*_ is the carbon emission (kgCO_2_e) in the production stage of building materials; n is the number of types of building materials; EF_i_ denotes the carbon emissions factor (kgCO_2_e/unit of material consumption) of type i building materials; M_i_ is the consumption of building materials of category i. The carbon emissions from the production process of building materials for prefabricated construction are the sum of carbon emissions released during the production of prefabricated floor slabs and prefabricated wall panels. According to Huang, the concrete consumed per cubic meter of precast floor slab is 512.401 cubic meters and 89.732 tonnes of steel reinforcement. According to the research of Shen^64^, the carbon emission factor of East China in 2023 was calculated to get 201.411 kg of carbon emission from the production stage of a single prefabricated wall panel, and the amount of floor and wall panels was calculated according to the volume of the hotel building, and then further calculated according to the carbon emission therefore.

7 $$\begin{array}{*{20}c} {Y_{2} = \mathop \sum \limits_{i = 1}^{n} V_{i} \times D_{i} \times X_{i} \times K} \\ \end{array}$$

where *Y*_2_ is the carbon emission (kgCO_2_e) in the transport phase of building materials; *i* = 1,2,…,*n* represents different categories of prefabricated components; *V*_*i*_ represents the total volume of the ith component; *D*_*i*_ represents the average transport distance of the *i*th component; *X*_*i*_ represents the carbon emission factor of the *i*th component per unit volume and per unit distance; *K* is the no-loading coefficient taken as 1.67. The carbon emission factor *X*_*i*_ refers to Cao’s^65^ study: 0.1234 kg/m^3^-km for prefabricated floor slabs and 0.0981 kg/m^3^-km for prefabricated walls. The transport distance of prefabricated components is 150 km according to the one-way transport distance of different cities.

8 $$\begin{array}{*{20}c} {Y_{3} = \mathop \sum \limits_{i = 1}^{n} T_{i} \times R \times E_{d} } \\ \end{array}$$

where *Y*_3_ denotes the carbon emission (kgCO_2_e) in the construction stage; *Ti* denotes the number of shifts spent on lifting the *i*th prefabricated component per unit volume; *R* denotes the energy consumption per unit shift; *Ed* is the carbon emission factor for electricity. The carbon emission in the construction stage of prefabricated building mainly refers to the carbon emission generated by the energy consumption of construction machinery during the on-site installation of prefabricated components. The carbon emission per unit volume in construction and installation stage refers to Cao’s^65^ study: 10.54 kg/m^3^ for prefabricated wall panels and 8.78 kg/m^3^ for prefabricated floor panels.

#### Decoupling calculation

The concept of ‘decoupling’ initially emerged from the field of physics and was subsequently incorporated into environmental economics^66^. It describes the process of reducing the dependency between environmental stress and economic growth, allowing resource consumption to become less constrained by economic development levels. In this study, decoupling analysis is conducted using the Tapio decoupling model, which employs derived elasticity coefficients and statistical relationships to assess the degree of decoupling between economic growth and environmental impact. However, the results of the Tapio model may be influenced by variations in data inputs and underlying assumptions. Future research could refine this analysis by integrating uncertainty assessments and scenario-based modeling to validate the stability of the decoupling trends. Utilizing the Tapio decoupling index model, this paper adopts the model linking building carbon emissions and economic growth as constructed by Qi^1^ to assess the decoupling status between carbon emissions and economic growth of star-rated hotels within the Hangzhou city area. present the overall decoupling status of hotels in Hangzhou regions from 2018 to 2023.

According to Tapio’s decoupling model, the decoupling relationship can be categorized into eight distinct stages based on the elasticity coefficient, as detailed in Table 1.

**Table 1:** Decoupling state type.

Decoupling state	ΔCO_2_/CO_2_	ΔG/G	Elasticity e	
Negative decoupling	> 0	> 0	e ≥ 1.2	Expansion negative decoupling
	> 0	< 0	< 0	Strong negative decoupling
	< 0	< 0	0 ≤ e < 0.8	Weak negative decoupling
Decoupling	> 0	> 0	0 ≤ e < 0.8	Weak decoupling
	< 0	> 0	< 0	Strong decoupling
	< 0	< 0	e ≥ 1.2	Recession decoupling
Connection	> 0	> 0	0.8 ≤ e < 1.2	Expansive coupling
	< 0	> 0	0.8 ≤ e < 1.2	Recessive coupling

Employing Qi’s^67^ model for economic and carbon emissions in the construction industry, this study explores the decoupling between hotel carbon emissions and revenue across two construction methods: conventional transmission building and modern prefabricated construction. The change in carbon emissions is the difference between the carbon emissions of conventional construction and prefabricated construction. The hotel economic data is based on statistical data from Hangzhou’s cultural and tourism big data, which is calculated by hotel room rates, occupancy rates, and days, and is statistically analyzed by region. Our aim is to evaluate the comparative effectiveness of these methods in reducing carbon emissions in the hotel sector while ensuring economic sustainability. The decoupling relationship between carbon dioxide emissions and energy consumption is quantified using the following formula:

9 $$\begin{array}{*{20}c} {\varepsilon_{2} \left( {L\left( {CO_{2} } \right),W\left( {EC} \right)} \right) = \frac{{\Delta L\left( {CO_{2} } \right)/L\left( {CO_{2} } \right)}}{{\Delta W\left( {EC} \right)/W\left( {EC} \right)}}} \\ \end{array}$$

where ε_2_(L(CO_2_), W(EC)) represents the decoupling elasticity between CO_2_ emissions and energy consumption, W(EC) is the energy consumption within the industry and L(CO_2_) is the carbon dioxide emissions.

10 $$\begin{array}{*{20}c} {\varepsilon_{3} \left( {W\left( {EC} \right),R\left( {GDP} \right)} \right) = \frac{{\Delta W\left( {EC} \right)/W\left( {EC} \right)}}{{\Delta R\left( {GDP} \right)/R\left( {GDP} \right)}}} \\ \end{array}$$

where ε_3_(W(EC), R(GDP)) represents the decoupling elasticity between energy consumption and the industry’s total output value, W(EC) is the industry’s energy consumption and R(GDP) is the industry’s total output value.

11 $$\begin{aligned} \varepsilon_{1} \left( {L\left( {CO_{2} } \right),R\left( {GDP} \right)} \right) & = \varepsilon_{2} \left( {L\left( {CO_{2} } \right),W\left( {EC} \right)} \right) \times \varepsilon_{3} \left( {W\left( {EC} \right),R\left( {GDP} \right)} \right) \\ & = \left( {\frac{{\Delta L\left( {CO_{2} } \right)/L\left( {CO_{2} } \right)}}{{\Delta W\left( {EC} \right)/W\left( {EC} \right)}}} \right) \times \left( {\frac{{\Delta W\left( {EC} \right)/W\left( {EC} \right)}}{{\Delta R\left( {GDP} \right)/R\left( {GDP} \right)}}} \right) \\ \end{aligned}$$

where ε_1_(L(CO_2_),R(GDP)) represents the decoupling elasticity the decoupling elasticity between CO_2_ emissions and the construction industry’s total output value, L(CO_2_) is the carbon dioxide emissions, W(EC) is the industry’s energy consumption and R(GDP) is the industry’s total output value.

### Data processing

This study begins by conducting a comprehensive search for all hotels in Hangzhou city on the Ctrip platform. Using the Octopus web-crawling software, data were extracted, including hotel latitude and longitude, number of rooms, star rating, administrative division, and room rates. A total of 1385 data points were collected, and after data cleaning, 1347 valid data points remained. The data cleaning process involved identifying and removing duplicate entries, filtering out incomplete or inconsistent records, and cross-checking star ratings and administrative divisions against official hotel directories where possible. To supplement the dataset, information on hotel room types, room type ratios, and average common areas of hotel rooms was referenced from industry data and related literature^68,69^. Economic data for hotels were primarily sourced from the Hangzhou Culture and Tourism Big Data website and Ctrip.com. Room rates were based on weekday prices in 2024, while occupancy rates were derived from public data on star-rated hotels across various districts and counties, as provided by the Culture and Tourism Big Data website. Although web-crawled data may introduce risks of inconsistencies, efforts were made to minimize inaccuracies by ensuring that extracted data aligned with publicly available industry reports and official sources. Future research could enhance data reliability by incorporating additional validation methods, such as cross-referencing multiple data sources or conducting field surveys.

To assess the carbon-saving impact of hotel buildings following the introduction of prefabricated technology, we measured carbon emissions under two construction modes using a carbon emission calculation model during the materialization phase. The total carbon emissions and the ratios λ_1_ and λ_2_ were analyzed to summarize the carbon-saving benefits of prefabricated technology. Here, λ_1_ represents the ratio of carbon emissions between the prefabricated mode and the conventional mode, while λ_2_ signifies the ratio of the change in carbon emissions to the conventional mode’s emissions. Further analysis was conducted to determine the degree of influence that different construction modes have on the carbon emissions of various star-rated hotels in Hangzhou during the materialization phase. Additionally, the decoupling relationship between carbon emissions and hotel turnover from 2018 to 2023 was examined to identify regions and star-rated hotels with the most significant carbon-saving effects. Based on these findings, recommendations for the implementation of prefabricated technology interventions were proposed.

## Results

### Hotel star levels impact on carbon emission differences between conventional and prefabricated construction

**Fig. 2:**
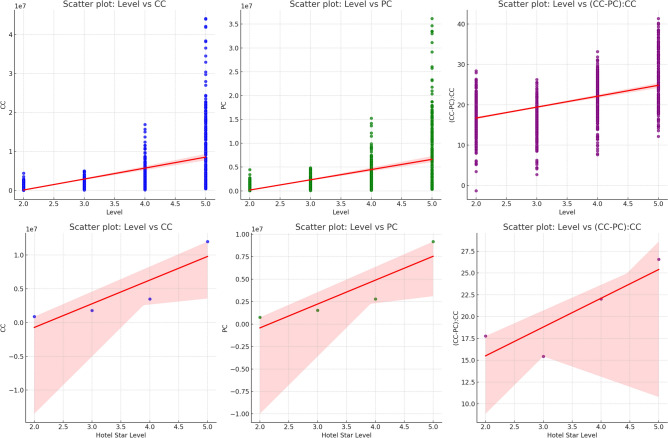
Conventional construction star-rated hotel regional carbon emission statistics.

The scatter plot (Fig. 2) depicting the relationship between hotel star level (Level) and the difference between conventional construction (CC) and prefabricated construction (PC) carbon emissions, denoted as (CC–PC):CC, offers valuable insights into how the two construction methods contribute to the overall carbon footprint across different hotel star levels. The (CC–PC):CC metric represents the difference in carbon emissions between Conventional Construction (CC) and Prefabricated Construction (PC), relative to the CC emissions. A higher value suggests that the Prefabricated Construction method is more carbon-efficient compared to Conventional Construction, while a lower value indicates the opposite. Higher Star Levels (4-star and 5-star hotels): The scatter plot indicates that higher-star hotels tend to have larger values of (CC–PC):CC, implying that the more luxurious hotels generally exhibit a larger difference between the two construction methods. This suggests that Prefabricated Construction might be more commonly implemented in higher-tier hotels, potentially due to cost-efficiency or modern construction practices that focus on minimizing carbon emissions. Mid-tier and Lower Star Levels (2-star and 3-star hotels): The trend in the scatter plot shows that mid-tier (3-star) and lower-tier (2-star) hotels exhibit smaller or even negative values for (CC–PC):CC, suggesting that these hotels, which might be less modern or more resource-constrained, may either have similar or less-efficient outcomes in terms of carbon emissions between the two construction methods. This could reflect a reliance on Conventional Construction methods, which may result in higher carbon emissions due to less advanced building techniques and older construction practices.

The scatter plot illustrates that, on average, (CC–PC):CC values are positive, meaning Prefabricated Construction is typically associated with lower carbon emissions compared to Conventional Construction. However, this difference is more pronounced in higher-tier hotels, which may have more resources to invest in sustainable construction technologies. The scatter plot suggests that higher-star hotels are more likely to adopt Prefabricated Construction as a means to reduce their overall environmental footprint, as the method tends to offer better carbon efficiency. In contrast, mid-tier and lower-star hotels may benefit from incorporating Prefabricated Construction techniques to improve their carbon efficiency and reduce reliance on higher-emission Conventional Construction methods.

The positive slope in the scatter plot indicates a trend where hotels with higher star levels show a greater difference in carbon emissions between CC and PC, confirming that more luxurious hotels are more likely to incorporate sustainable construction methods. The variability within each star level group suggests that factors such as building size, location, and other construction practices might also influence the (CC–PC):CC values. To optimize carbon emissions, mid-tier and lower-tier hotels could explore the adoption of Prefabricated Construction to reduce their environmental impact. The potential for energy savings and carbon footprint reduction could be significant in these segments. Additional factors, such as the adoption of renewable energy sources, building materials, and operational efficiency, could further explain the observed variability and refine our understanding of the carbon footprint associated with each hotel category.

### Clustering analysis of carbon emissions in conventional and prefabricated hotel construction

**Fig. 3:**
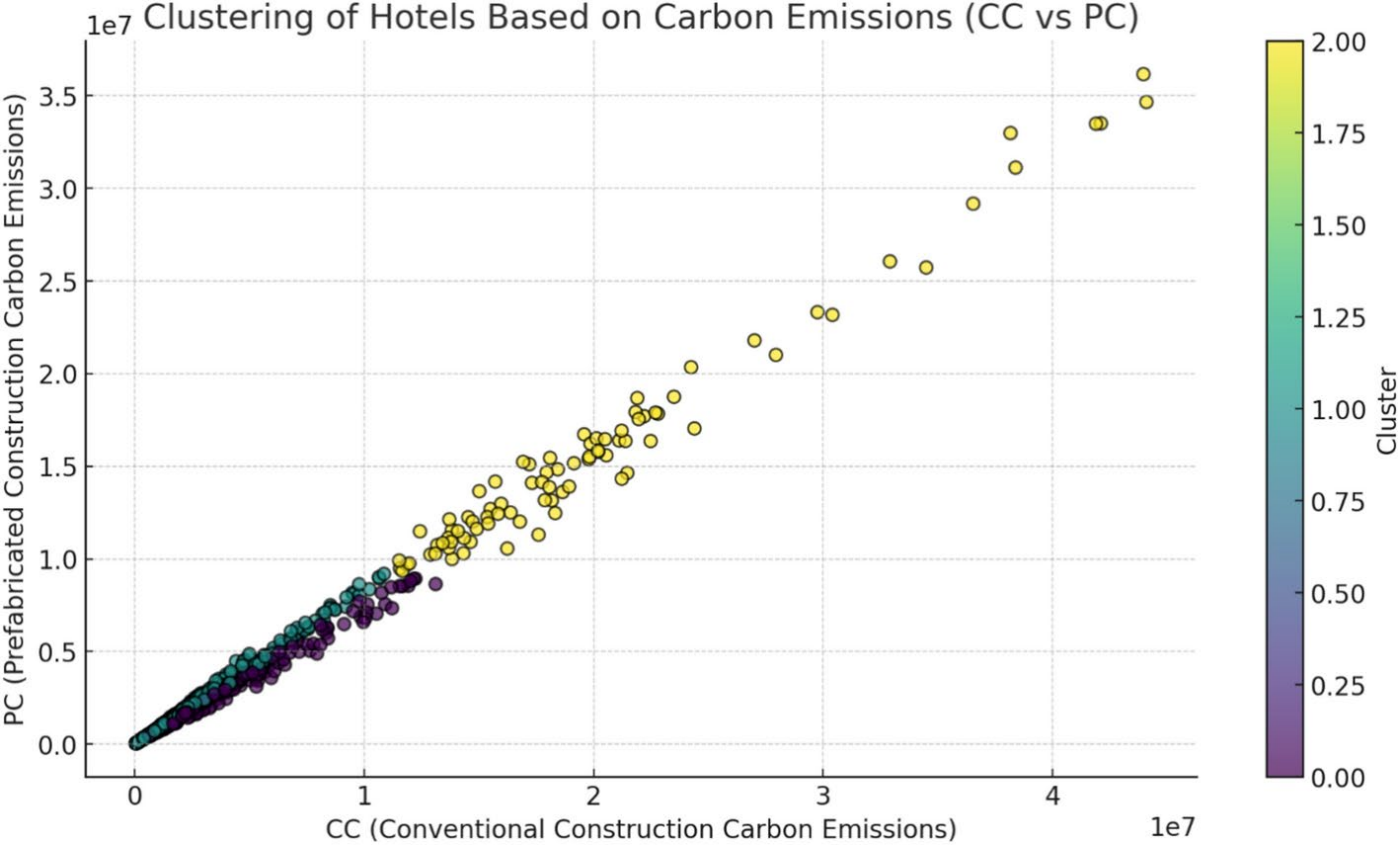
Clustering of Hotels Based on Carbon Emissions (CC vs. PC).

The clustering analysis (Fig. 3), based on the carbon emissions of hotels under Conventional Construction (CC) and Prefabricated Construction (PC), reveals key insights into how hotel star levels and construction methods contribute to their overall carbon footprint. Using the K-means clustering method, three distinct clusters were identified, each representing a group of hotels with similar carbon emission profiles.

The first cluster consists of hotels with low carbon emissions for both CC and PC, which are typically lower-tier establishments. The average CC and PC values for this cluster are 320,215.87 kgCO_2_ and 288,086.82 kgCO_2_, respectively, indicating that these hotels produce lower emissions overall. The negative or low values of the (CC–PC):CC metric suggest that these hotels rely more on Prefabricated Construction, which is more carbon-efficient than Conventional Construction. This can be attributed to their smaller scale and possibly older construction methods, which may have been optimized for efficiency. The second cluster shows moderate carbon emissions, with average CC and PC values of 361,740.95 kgCO_2_ and 285,640.27 kgCO_2_, respectively. The relatively balanced (CC–PC):CC values suggest that these mid-tier hotels, typically 3-star establishments, do not show a significant difference in carbon emissions between CC and PC. This reflects a more conventional approach to construction, where sustainable practices might be employed to a limited extent but are not yet a major factor in their design or construction processes. The third cluster includes high-end hotels (4-star and 5-star), which exhibit the highest carbon emissions. The average CC and PC values for this cluster are 481,856.86 kgCO_2_ and 386,906.64 kgCO_2_, respectively, indicating a higher carbon footprint than the other two clusters. The (CC–PC):CC values for this cluster are positive, suggesting that these hotels primarily use Conventional Construction methods, which have a higher carbon emission profile compared to Prefabricated Construction. Despite potential efforts to incorporate sustainable construction techniques, the larger scale and complexity of high-star hotels result in higher overall emissions.

The clustering analysis clearly shows a correlation between hotel star levels and carbon emissions. Higher-star hotels exhibit significantly higher emissions, which can be attributed to their larger size, more complex designs, and greater use of Conventional Construction methods. In contrast, lower-star hotels benefit from Prefabricated Construction due to its carbon efficiency, which results in lower carbon emissions. Mid-tier hotels demonstrate a moderate carbon footprint, with a balanced reliance on both CC and PC methods. These hotels might benefit from adopting more advanced Prefabricated Construction techniques to further reduce emissions and improve overall sustainability. The positive and negative trends observed in the (CC–PC):CC metric highlight the potential for sustainable construction practices to significantly reduce carbon emissions, especially in mid-tier and higher-tier hotels. The findings suggest that adopting more energy-efficient construction methods could provide long-term environmental and economic benefits across the hotel industry.

### Spatial temporal decoupling trends of hotel carbon emissions (2018–2023)

**Fig. 4:**
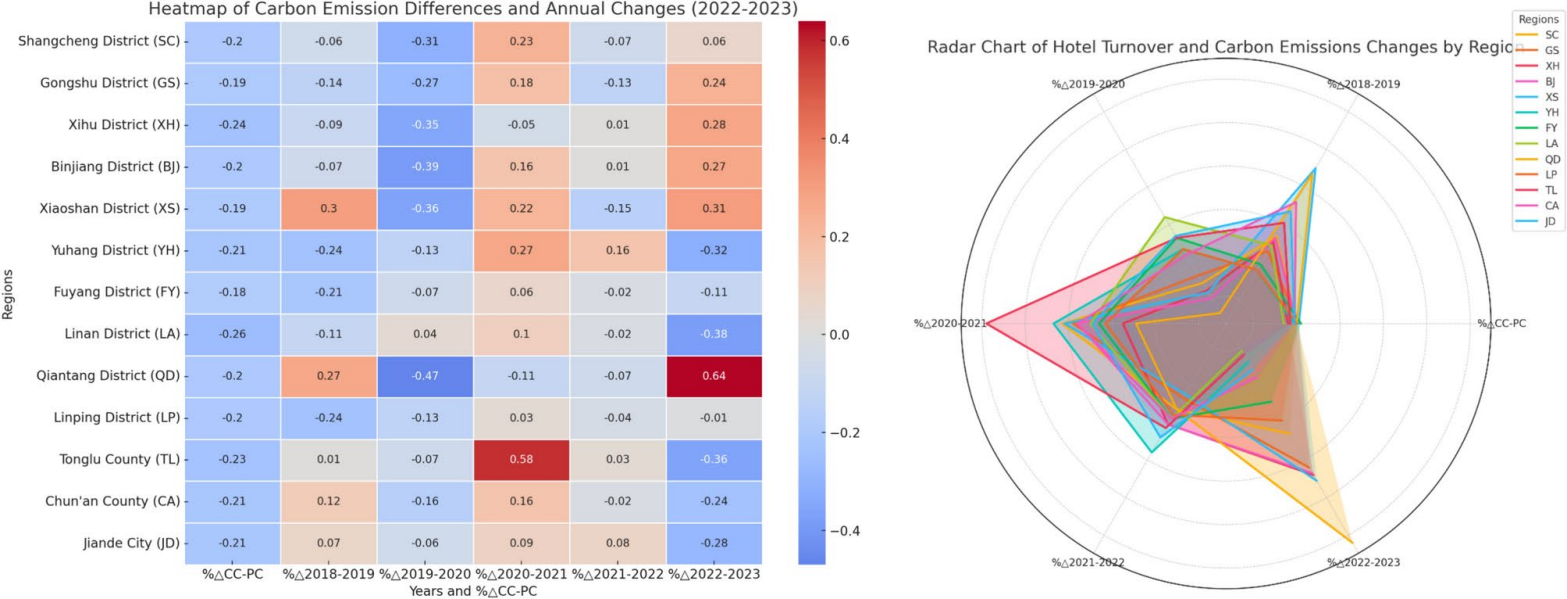
Driving factors decoupling elastic changes of regional hotel carbon emissions from 2018 to 2023.

Between 2018 and 2023, the hotel sector in Hangzhou exhibited regionally distinct turnover trends (Fig. 4), significantly influenced by the COVID-19 pandemic and the Asian Games. The pandemic caused a general downturn in hotel turnover across the city, with a temporary increase in remote suburbs, while the Asian Games led to a notable turnover boost in central areas and a downturn in the suburbs. In 2020, the pandemic sharply reduced turnover, especially in uptown, West Lake, Binjiang, Xiaoshan, and Qiantang Districts, where declines exceeded 30%, and Gongshu District, which saw a drop of over 20%. In 2021, hotel turnover rebounded, with uptown, Xiaoshan, Yuhang, and Tonglu seeing increases of over 20%. Other regions experienced gradual growth, except for West Lake, which continued to decline, and Tonglu County, which saw a remarkable rise of over 50%, as tourists sought short-distance travel to avoid crowded areas. In 2022, turnover fluctuated modestly, with a general downward trend. In 2023, turnover surged in uptown, Xihu, Binjiang, Gongshu, and Qiantang districts, with uptown seeing an increase of over 20%, and Qiantang’s turnover spiking by more than 60% due to the lifting of pandemic restrictions and the influx of tourists for the Asian Games. Conversely, remote suburbs experienced a decline of over 20%, as the end of pandemic restrictions shifted travel preferences toward inter-provincial long-distance trips. This analysis highlights the varying impacts of the pandemic and the Asian Games on hotel turnover across different regions of Hangzhou.

**Fig. 5:**
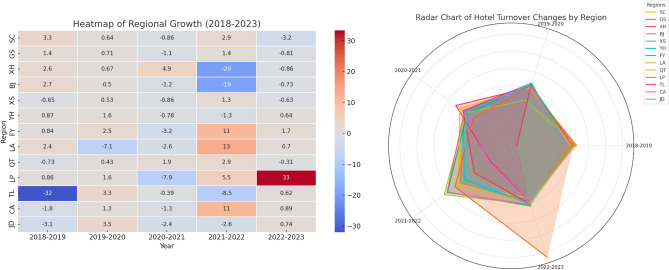
The decoupling index exhibits.

Between 2018 and 2023, the introduction of prefabricated technology in Hangzhou’s hotel sector led to distinct decoupling trends (Fig. 5) in economic growth and environmental impact. Strong decoupling occurred primarily between 2020 and 2021, where economic growth was accompanied by a decline in environmental pressure, largely due to the pandemic, which reduced hotel occupancy rates and, consequently, hotel carbon emissions. This decline in emissions was not due to operational changes but the reduced activity caused by pandemic restrictions. Weak negative decoupling, where both carbon emissions and turnover declined but emissions decreased at a slower rate, was observed in 2019–2020 and 2022–2023. The core areas of Hangzhou and Qiantang District were heavily impacted by the pandemic, with sharp declines in hotel occupancy rates. After the pandemic, regions like Yuhang and Tonglu experienced weak decoupling due to an initial surge in tourist numbers followed by a post-pandemic drop. Recession decoupling, where both hotel carbon emissions and turnover declined but turnover decreased at a slower rate than emissions, occurred throughout the study period, particularly in remote suburbs and districts like Yuhang and Fuyang, which were less affected by the pandemic. These regions saw reduced tourism but were less impacted than core areas. As the pandemic subsided, turnover in some areas recovered, but West Lake and Qiantang districts continued their downward trends, indicating recession decoupling. In the late pandemic stage and during the Asian Games, while turnover in the core areas increased, the suburbs continued to show a decline, with Fuyang and Linping districts experiencing slow declines. Recessive coupling, where carbon emissions and hotel turnover declined at similar rates, was observed only during two periods: 2018–2019 and 2022–2023. The first phase occurred in districts like Fuyang, Linping, and Yuhang, which were less affected by the pandemic, while the second phase occurred in Chun’an County. This analysis highlights how the impact of the pandemic and the adoption of prefabricated technology influenced the hotel sector’s carbon emissions and turnover in Hangzhou, with varying regional responses.

### Carbon emissions between prefabricated and conventional construction

The carbon emission patterns of star-rated hotels exhibit consistency across different administrative regions following the adoption of prefabricated construction technology. Total Carbon Emissions: Higher-star hotels generate significantly greater carbon emissions compared to lower-star hotels, primarily due to their larger scale and increased material consumption. Stability of Carbon Reduction Effects: The variability in carbon emissions among two-star hotels in Hangzhou is less pronounced than that observed in three-star hotels, indicating that the carbon reduction effect is more stable in two-star hotels after the integration of prefabricated technology. This stability is evident across the key phases of material production, transportation, and on-site construction following the implementation of prefabrication. Across all construction phases within Hangzhou, high-star hotels consistently emit more carbon than low-star hotels. Furthermore, the fluctuation in carbon emissions for two-star hotels remains lower than that for three-star hotels, reinforcing the conclusion that prefabricated technology facilitates a more stable reduction in carbon emissions in lower-tier hotels. Comparison of Conventional and Prefabricated Construction: With the exception of the material transportation phase, conventional construction results in significantly higher carbon emissions than prefabricated construction across all star-rated hotel categories in the other two phases. Impact of the COVID-19 Pandemic on Carbon Decoupling: The pandemic influenced carbon decoupling trends in Hangzhou’s hotel sector, with the strongest decoupling effect observed in 2021 and the weakest in 2022. Spatial Analysis: The pandemic’s impact on carbon decoupling varied across Hangzhou’s core, peri-urban, and far-urban areas. While core and peri-urban areas generally experienced positive effects on carbon decoupling, the magnitude of impact differed across regions, whereas peri-urban areas encountered a negative influence, impeding sustainable recovery. Total Carbon Emissions in the Materialization Phase: Among all hotel categories, two-star hotels demonstrated the most effective carbon reduction following the implementation of prefabricated technology. Regional Prioritization for Carbon Reduction: Considering emission reduction strategies across different regions, priority should be given to the material production and on-site construction phases, as these stages present the greatest potential for carbon mitigation in the near future.

## Discussion

This study offers a comprehensive analysis of carbon emission trends in star-rated hotels across Hangzhou, examining both prefabricated and conventional construction methodologies. The findings underscore the significant impact of construction technology on carbon emissions, revealing distinct patterns when analyzed by region and hotel star level. The study confirms that total carbon emissions in the materialization phase of prefabricated construction are generally lower than those in conventional construction across all hotel categories, regardless of star ratings. This suggests that implementing prefabricated technology can yield substantial environmental benefits, particularly during the building material production and transportation phases. While the adoption of prefabricated construction methods demonstrates greater carbon efficiency in higher-star hotels, it is clear that the environmental benefits are not uniformly distributed across regions. For instance, uptown areas and high-star hotels show a clear reduction in carbon emissions with the rise in star ratings, whereas districts such as Fuyang and Chun’an exhibit more complex trends, with fluctuations in emissions as star levels increase. These variations may be attributed to differences in regional development, hotel size, and specific construction methods.

Additionally, the study highlights that high-star hotels tend to have greater total carbon emissions than lower-star hotels, regardless of the construction method. This aligns with the understanding that larger, more luxurious hotels require more resources and generate higher emissions due to their complex designs and operational demands. Interestingly, the carbon-saving effects of prefabricated technology were found to be more stable in lower-star hotels, particularly two-star hotels, suggesting that smaller hotels may benefit more consistently from prefabricated construction in terms of carbon emission reduction. In contrast, mid-tier and high-tier hotels exhibit more variability in their emissions, likely due to the larger scale and operational complexity of these establishments. The clustering analysis further emphasizes the correlation between hotel star levels and carbon emissions, with high-end hotels (4-star and 5-star) exhibiting the highest emissions due to their size and reliance on conventional construction methods, while lower-tier hotels benefit from more carbon-efficient prefabricated construction. This indicates that integrating sustainable construction practices, such as prefabricated technology, could offer significant environmental benefits, particularly for mid-tier and higher-tier hotels.

The study also emphasizes the role of external factors, particularly the COVID-19 pandemic, on carbon decoupling trends in Hangzhou’s hotel sector. The pandemic led to strong decoupling in 2020–2021, where economic growth was accompanied by a reduction in carbon emissions, largely due to a decline in hotel occupancy rates. In contrast, weaker and recessionary decoupling patterns emerged in 2022–2023, as the economy began to recover, but emissions continued to decline at a slower rate. The varying impacts of the pandemic across different districts, especially core, peri-urban, and suburban areas, suggest that the ability to decouple economic growth from carbon emissions is highly context-dependent. Core areas, where tourism and hotel occupancy were significantly impacted, exhibited a more pronounced decoupling effect compared to the suburbs, where recovery was slower.

This study has several limitations. First, the analysis focuses on carbon emissions from the materialization phase, considering only steel, concrete, and masonry for Conventional Construction, excluding other materials like insulation and glazing. While this simplifies the comparison of primary structural components, it limits the scope of the study. Future research should include a broader range of materials to better capture the full carbon footprint of Conventional Construction. For prefabricated construction, the assumption of a 100% prefabrication rate isolates its impact, focusing on prefabricated floor slabs and wall panels. Although this provides an upper boundary for potential carbon reductions, it may not reflect real-world construction diversity. Future studies could incorporate hybrid construction methods and a wider range of materials to improve applicability. Transport distances, based on industry practices, are assumed to be 30 km for local and 150 km for intercity transport, with potential inaccuracies due to the lack of specific data for Hangzhou’s prefabricated component logistics. Future research could improve accuracy by incorporating empirical transport data. The use of the Tapio decoupling model provides valuable insights, but its results are influenced by data inputs and assumptions. Future studies could integrate uncertainty assessments and scenario-based modeling to refine decoupling analysis. Finally, although data were cleaned and cross-checked from the Ctrip platform, web-crawled data may introduce inconsistencies. Future research should cross-reference multiple data sources or conduct field surveys to improve data reliability. These limitations suggest areas for future refinement, particularly in material diversity, transport logistics, and data validation.

## Conclusion

This study provides a comprehensive assessment of carbon emissions in star-rated hotels in Hangzhou, focusing on the comparative analysis of Prefabricated Construction (PC) and Conventional Construction (CC) methodologies. The findings demonstrate that Prefabricated Construction generally results in lower carbon emissions compared to Conventional Construction, particularly in the materialization phase. Distinct patterns in carbon emissions were observed across various administrative regions and hotel star levels, with higher-star hotels exhibiting greater total emissions, while lower-star hotels showed more consistent carbon-saving benefits from Prefabricated Construction. The clustering analysis further emphasized the correlation between hotel star levels and carbon emissions, with high-star hotels showing higher emissions due to larger scale and reliance on Conventional Construction, while lower-tier hotels benefit more from the adoption of Prefabricated Construction. The study also highlighted the influence of external factors such as the COVID-19 pandemic, which temporarily reduced emissions due to decreased hotel occupancy, particularly in core urban areas. However, decoupling trends varied by region and star level, with core areas seeing a more pronounced decoupling effect. This research underscores the importance of integrating sustainable construction technologies like Prefabricated Construction to mitigate the environmental impact of the hotel sector, especially in mid-tier and lower-tier hotels. Future research should expand the scope to include a wider range of materials, refine transport logistics assumptions, and incorporate real-world data to enhance the applicability of findings to diverse construction projects. Moreover, integrating renewable energy sources and assessing lifecycle carbon emissions could further improve sustainability within the sector.

## Data Availability

The datasets used and analysed during the current study available from the corresponding author on reasonable request.
