# Correction: Prefabricated building construction in materialization phase as catalysts for hotel low-carbon transitions via hybrid computational visualization algorithms

**DOI:** 10.1038/s41598-025-03815-w

**Published:** 2025-06-03

**Authors:** Gangwei Cai, Xiaoting Guo, Yuguang Sun

**Affiliations:** 1 College of Architecture and Urban Planning, Tongji University, Shanghai, 200092 China; 2 Hangzhou International Urbanology Research Center & Zhejiang Urban Governance Studies Center, Hangzhou, 311121 China; 3 Baoye Daiwa Industrialized House Manufacturing Co., Ltd., Daiwa House Group, Osaka, 530-8241 Japan; 4 Baoye Group Company Limited, Shaoxing, 312030 China; 5 School of Civil Engineering and Architecture, Zhejiang University of Science and Technology, Hangzhou, 310023 China

Correction to: *Scientific Reports* 10.1038/s41598-025-92200-8, published online 05 March 2025

The original version of this Article contained an error in the name of the author Yuguang Sun, which was incorrectly given as Yuguang Su.

The original Article has been corrected.

